# Mixed formulation of mRNA and protein‐based COVID‐19 vaccines triggered superior neutralizing antibody responses

**DOI:** 10.1002/mco2.188

**Published:** 2022-12-02

**Authors:** Jialu Zhang, Qian He, Xujia Yan, Jianyang Liu, Yu Bai, Chaoqiang An, Bopei Cui, Fan Gao, Qunying Mao, Junzhi Wang, Miao Xu, Zhenglun Liang

**Affiliations:** ^1^ Division of Hepatitis and Enterovirus Vaccines, Institute of Biological Products, National Institutes for Food and Drug Control NHC Key Laboratory of Research on Quality and Standardization of Biotech Products, NMPA Key Laboratory for Quality Research and Evaluation of Biological Products Beijing People's Republic of China

**Keywords:** COVID‐19 vaccine, heterologous prime‐boost, neutralizing antibody, SARS‐CoV‐2, Th1 response

## Abstract

Integrating different types of vaccines into a singular immunization regimen is an effective and accessible approach to strengthen and broaden the immunogenicity of existing coronavirus disease 2019 (COVID‐19) vaccine candidates. To optimize the immunization strategy of the novel mRNA‐based vaccine and recombinant protein subunit vaccine that attracted much attention in COVID‐19 vaccine development, we evaluated the immunogenicity of different combined regimens with the mRNA vaccine (RNA‐RBD) and protein subunit vaccine (PS‐RBD) in mice. Compared with homologous immunization of RNA‐RBD or PS‐RBD, heterologous prime‐boost strategies for mRNA and protein subunit vaccines failed to simultaneously enhance neutralizing antibody (NAb) and Th1 cellular response in this study, showing modestly higher serum neutralizing activity and antibody‐dependent cell‐mediated cytotoxicity for “PS‐RBD prime, RNA‐RBD boost” and robust Th1 type cellular response for “RNA‐RBD prime, PS‐RBD boost”. Interestingly, immunizing the mice with the mixed formulation of the two aforementioned vaccines in various proportions further significantly enhanced the NAb responses against ancestral, Delta, and Omicron strains and manifested increased Th1‐type responses, suggesting that a mixed formulation of mRNA and protein vaccines might be a more prospective vaccination strategy. This study provides basic research data on the combined vaccination strategies of mRNA and protein‐based COVID‐19 vaccines.

## Graphical abstract

The immunogenicity of vaccines can be enhanced by the optimization of immunization strategies. In this paper, we investigated the immunogenicity of different combined regimens with the mRNA vaccine RNA‐RBD and protein subunit vaccine PS‐RBD. The result showed that compared with homologous immunization, heterologous prime‐boost strategies for mRNA and protein subunit vaccines failed to simultaneously enhance NAb and Th1 cellular response, but immunizing the mice with the mixed formulation of the two aforementioned vaccines in various proportions further significantly enhanced the NAb responses and Th1 cellular response.

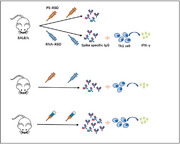

## 1 INTRODUCTION

The global epidemic of severe acute respiratory syndrome coronavirus 2 (SARS‑CoV‑2) began in 2019 and has been ongoing for more than 2 years. Vaccination has always been considered the most effective approach to eliminate the coronavirus disease 2019 (COVID‐19) epidemic, and nearly all vaccine technologies have been used for the development of an “effective and safe” COVID‐19 vaccine candidate.^1^ Forty‐nine vaccines^2^ have been approved for marketing or emergency using by National Regulatory Agencies across the world, and all demonstrated a vaccine efficacy >50% against the ancestral SARS‐CoV‐2 strain.^3^, ^4^, ^5^, ^6^, ^7^ However, the rapid viral mutation facilitates the SARS‐CoV‐2 variants to escape the vaccine‐induced immunity, leading to an increase in breakthrough infections in the vaccinated population.^8^, ^9^ It is urgently needed to further improve the immunogenicity of the existing COVID‐19 vaccine candidates against variants of concern (VOCs), so as to strengthen the immune defense of the population against severe infection.

A heterologous prime‐boost immunization strategy,^10^ wherein vaccines developed by different technology platforms or antigen sequences are administered sequentially, has been widely studied and proved to be effective in improving the immunogenicity and protection of existing vaccines, including those against HIV, influenza, polio, and hepatitis viruses.^11^, ^12^, ^13^, ^14^, ^15^ Since nearly all vaccine technologies have been used for COVID‐19 vaccine development, various heterologous prime‐boost regimens, such as mRNA and adenovirus‐vectored vaccines,^16^, ^17^, ^18^ mRNA and inactivated virus vaccines,^19^, ^20^ adenovirus‐vectored and inactivated virus vaccines,^21^ inactivated virus and recombinant protein vaccines,^22^ have been investigated in animal models^22^ and clinical trials.^16^, ^17^, ^18^, ^19^, ^20^, ^21^ The aforementioned heterologous regimens all showed to be superior or equivalent to a single vaccine regimen in immunogenicity and had a good safety profile. In addition, mixed formulation or co‐delivery of certain COVID‐19 vaccines based on distinct platforms could also elevate the protective immune responses.^23^

Among the COVID‐19 vaccines under clinical trials, mRNA and recombinant protein vaccines are the two commonly studied platforms and account for 23% and 32% according to World Health Organization collected data.^24^ In particular, the mRNA vaccine could induce potent humoral and cellular immune responses at the same time, conferring a potential to be applied for clinical use.^25^ As members of the firstly marketed generation of COVID‐19 vaccines, mRNA vaccines such as BNT162b2^26^ and mRNA1273^27^, and recombinant protein vaccines such as ZF2001^28^ and NVX‐2373^29^, raised global concern about the potential protection ability against SARS‐CoV‐2 VOCs. A proper combined application strategy of mRNA and protein subunit COVID‐19 vaccines might be an effective and achievable means to better cope with the wide‐spreading epidemic. However, there have been few studies on the combined application of mRNA and recombinant protein vaccines.^30^ Li et al.^23^ have confirmed that heterologous prime‐boost or co‐delivery of DNA and protein vaccines could effectively improve the neutralizing antibody (NAb) responses. However, it's hard to extend the conclusion to mRNA vaccines, due to significant differences between DNA and mRNA vaccines in the process of antigen expression and presentation, as well as the immunity induction. More detailed research and discussion on immunization strategies of mRNA and protein vaccines are needed and might assist in the formulation of further vaccination policies. In this study, BALB/c mice were immunized with recombinant protein subunit vaccine (PS‐RBD) and RBD‐expressing mRNA vaccine (RNA‐RBD) by homologous, heterologous, and mixed formulation vaccination strategies. The different combined regimens were tested and evaluated, and the particular role of the mRNA vaccine in a combined immunization regimen was deeply discussed.

## 2 RESULTS

### 2.1 Binding antibody responses elicited by homologous and heterologous immunization with PS‐RBD and RNA‐RBD in mice

**FIGURE 1:**
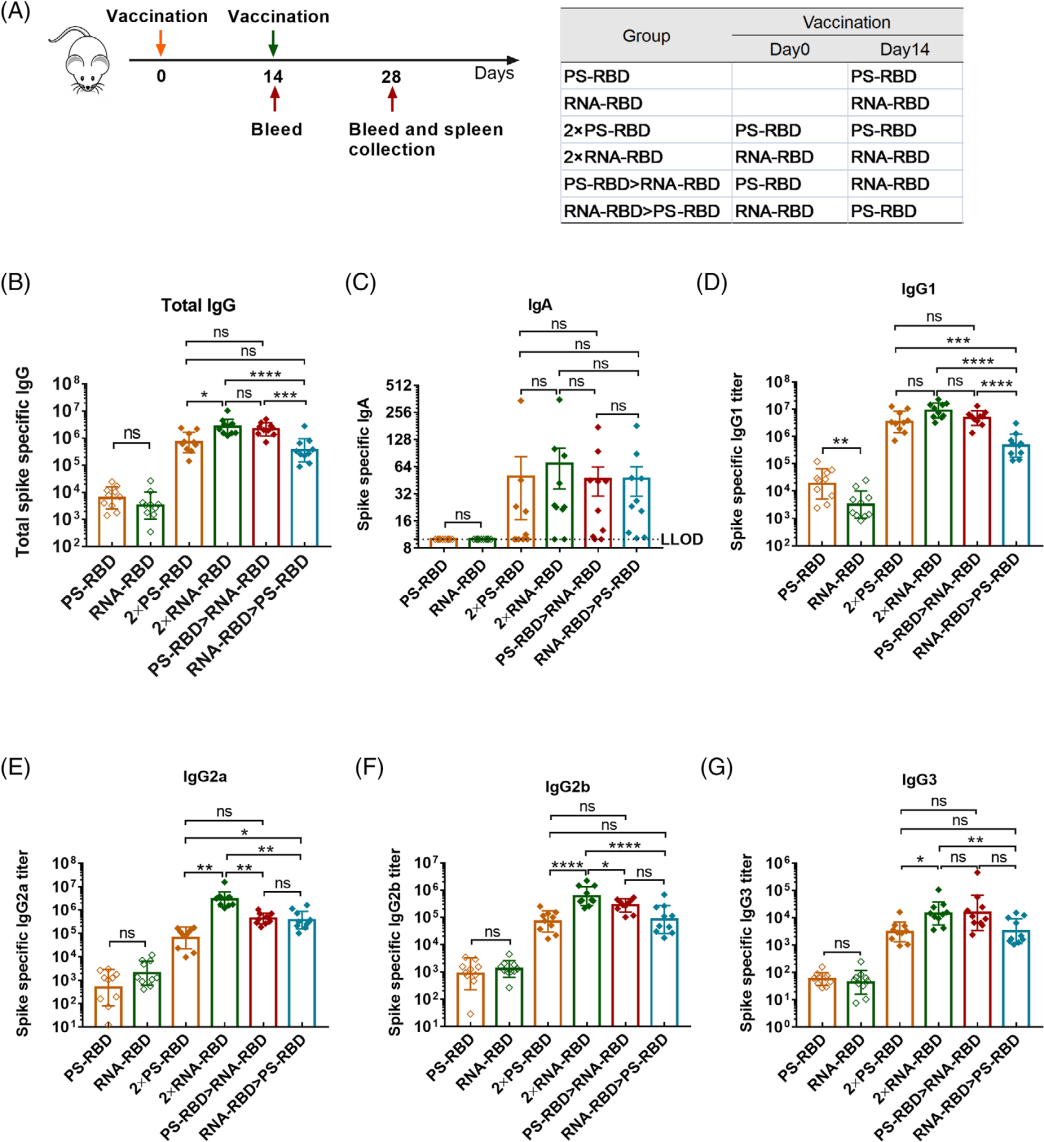
**Humoral response and IgG subtype induced by homologous and heterologous prime‐boost with PS‐RBD and RNA‐RBD in mice. (A)**. Schematic representation of experimental protocol. Mice in six groups were immunized with different vaccine regimens: PS‐RBD, RNA‐RBD, 2 × PS‐RBD, 2 × RNA‐RBD, PS‐RBD > RNA‐RBD, and RNA‐RBD > PS‐RBD. N = 10 per group. For all mice, blood was collected on days 14 and 28. **(B–G)**. Spike‐specific immunoglobulin G (IgG) (B), IgA (C), and IgG subtypes (D–G) titers were measured by enzyme‐linked immunosorbent assay (ELISA). LLOD: lower limit of detection. *N* = 10 per group, one spot represents one sample. Bars represent the geomean ± geometric SD, ns: *p* > 0.05, *p < 0.05, **p < 0.01, ***p < 0.001, ****p < 0.0001

To define a superior immunization strategy for mRNA vaccine and protein subunit vaccine, an mRNA vaccine named RNA‐RBD and a recombinant protein subunit vaccine named PS‐RBD, both harboring an ancestral SARS‐CoV‐2 receptor binding receptor (RBD) as the specific antigen, were tested for homologous and heterologous prime‐boost immunization. To be specific, mice were divided into six groups and immunized with different vaccines at 2 weeks interval as shown in Figure 1A: one single dose of PS‐RBD (PS‐RBD), one single dose of RNA‐RBD (RNA‐RBD), two doses of PS‐RBD (2 × PS‐RBD), two doses of RNA‐RBD (2 × RNA‐RBD), PS‐RBD prime and RNA‐RBD boost (PS‐RBD > RNA‐RBD), or RNA‐RBD prime and PS‐RBD boost (RNA‐RBD > PS‐RBD). The serum binding antibody for all groups was measured 2 weeks after immunization. For the total immunoglobulin G (IgG) titer, one dose of PS‐RBD (PS‐RBD) is about 1.92 folds of one dose of RNA‐RBD (RNA‐RBD). Whereas, after a homologous booster dose, the geometric mean titers (GMTs) of total IgG in the 2 × RNA‐RBD group was 2,673,607 (95% confidence interval [CI]: 1,819,70–3981.072) and significantly higher than that of the 2 × PS‐RBD groups (GMTs: 708,284, 95% CI 426,580–1,174,898) (*p* = 0.0169), indicating a stronger boosting potential of RNA‐RBD than PS‐RBD. For the IgG in heterologous prime‐boost groups, the IgG titers of the PS‐RBD > RNA‐RBD group showed to be significantly higher than that of the RNA‐RBD > PS‐RBD group, with GMTs of 2,152,747 (95% CI: 1,548,817–2,951,209) and 360,276 (95% CI: 204,174–645,654), respectively (*p* = 0.0005). However, there was no significant difference between PS‐RBD > RNA‐RBD and homologous 2 × RNA‐RBD or 2 × PS‐RBD (Figure 1B). For IgA, none IgA was detected in groups PS‐RBD and RNA‐RBD, and there was no significant difference among the four groups 2 × PS‐RBD (GMTs: 20, 95% CI: 10–38), 2 × RNA‐RBD (GMTs: 36, 95% CI: 19–68), PS‐RBD > RNA‐RBD (GMTs: 29, 95% CI: 16–52) and RNA‐RBD > PS‐RBD (GMTs: 30, 95% CI: 17–52) (Figure 1C).

Subsequently, IgG subtypes, like IgG1, IgG2a, IgG2b, and IgG3, were measured. The GMTs of IgG1 induced by one dose of PS‐RBD was 18,247 (95% CI: 8,511‐38,905) and significantly higher than that induced by one dose of RNA‐RBD (GMTs: 3,204, 95% CI: 1,660‐6,310; *p* = 0.0021). The groups PS‐RBD and RNA‐RBD showed no significant difference in IgG2a, IgG2b, and IgG3. Similar to total IgG, after the homologous booster dose, the IgG1 level of group 2 × RNA‐RBD increased and was similar to that induced by 2 × PS‐RBD, whereas, the IgG2a level of 2 × RNA‐RBD exceeded that induced by 2 × PS‐RBD. The titers of the four IgG subtypes induced by PS‐RBD > RNA‐RBD were higher than those of RNA‐RBD > PS‐RBD in varying degrees but were lower than those in the 2 × RNA‐RBD groups (Figure 1D–G).

**FIGURE 2:**
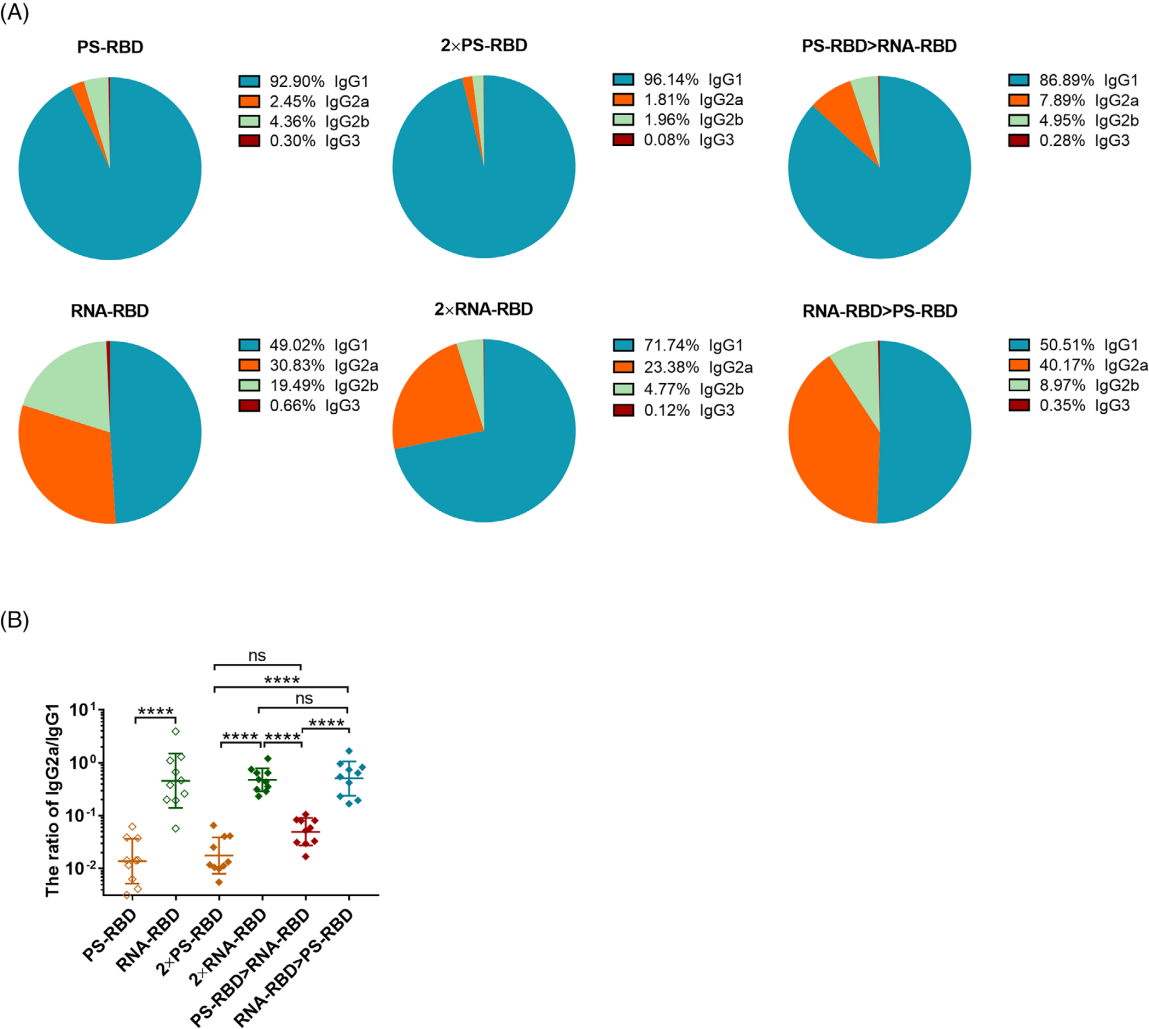
**The ratios of Serum** immunoglobulin G (IgG) **subtypes induced by homologous and heterologous immunization with PS‐RBD and RNA‐RBD in mice. (A,B)**. The constituent ratios of each IgG subtype and the ratios of IgG2a/IgG1 were calculated and shown in the pie (A) and scatter plots (B) chart. *N* = 10 per group, one spot represents one sample. Bars represent the mean ± SD, ns: *p* > 0.05, ****p < 0.0001. *p*‐Values were derived by Tukey's multiple comparisons test after the ratio of IgG2a/IgG1 was log‐transformed

Furthermore, by analyzing the constituent ratio of each IgG subtype in each group, we found that the IgG1 ratio in the groups with PS‐RBD as the primary vaccine (PS‐RBD, 2 × PS‐RBD, PS‐RBD > RNA‐RBD) reached >86%, while IgG2a only accounted for 2.45%, 1.81%, and 7.89%, respectively. In comparison, groups with RNA‐RBD as the primary vaccine (RNA‐RBD, 2 × RNA‐RBD, RNA‐RBD > PS‐RBD) had relatively lower ratios of IgG1 (49.02%, 71.74%, and 50.51%, respectively), while the ratios of IgG2a increased in varying degrees (30.83%, 23.38%, and 40.17%, respectively) (Figure 2A). The ratios of IgG2a/IgG1 were 0.02, 0.87, 0.02, 0.53, 0.06, and 0.64 for PS‐RBD, RNA‐RBD, 2 × PS‐RBD, 2 × RNA‐RBD, PS‐RBD > RNA‐RBD and RNA‐RBD > PS‐RBD, respectively (Figure 2B).

Taken together, RNA‐RBD induced a higher level of binding antibody titers following homologous booster compared to PS‐RBD. The heterologous prime‐boost immunization regimen didn't increase the IgG level of RNA‐RBD and PS‐RBD, but it regulated the constituent ratio of the IgG subtypes. RNA‐RBD priming induced higher ratios of IgG2a/IgG1.

### 2.2 Heterologous priming with PS‐RBD and boosting with RNA‐RBD improved the serum‐neutralizing activity in mice

**FIGURE 3:**
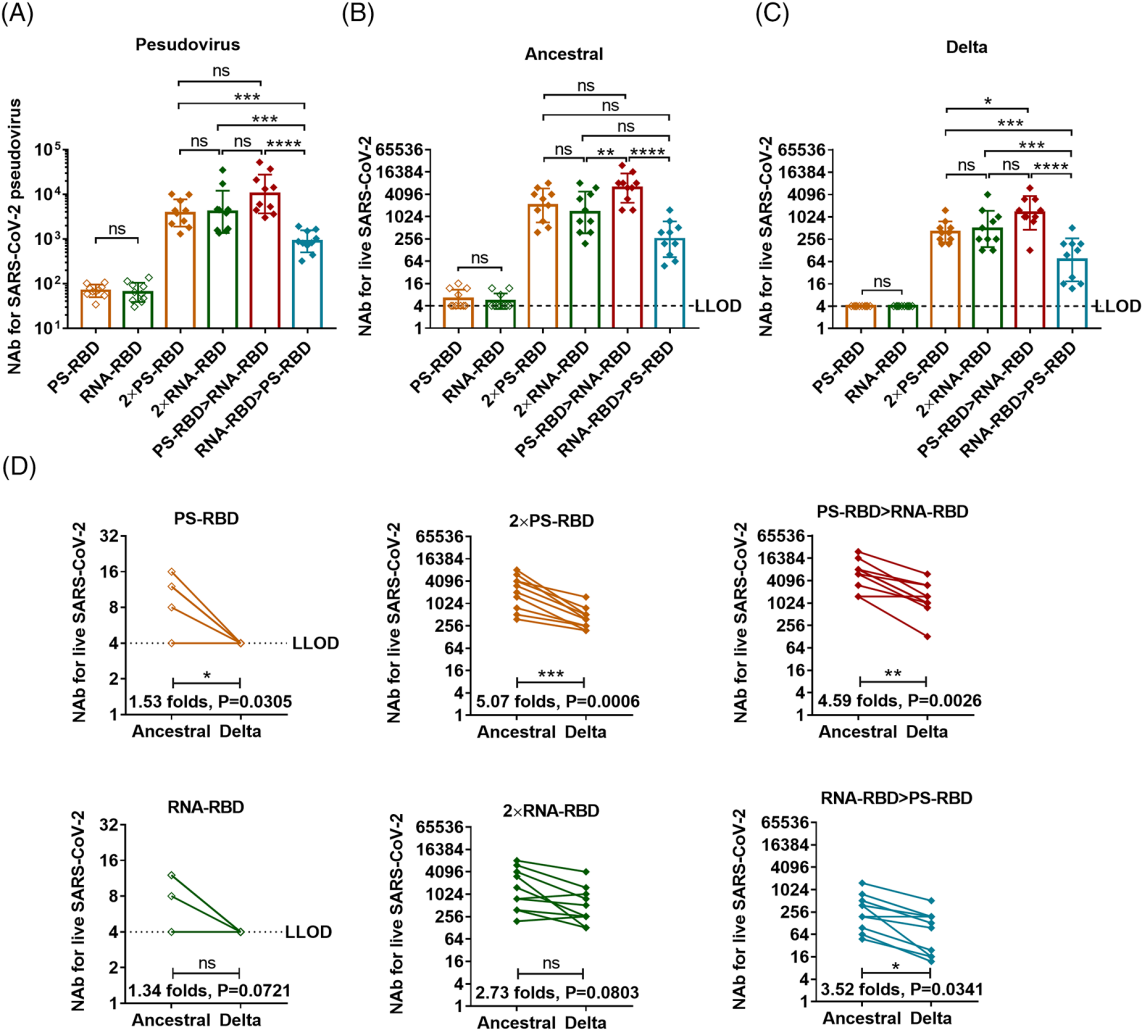
**Serum neutralizing activity induced by homologous and heterologous immunization with PS‐RBD and RNA‐RBD in mice. (A)**. Serum neutralizing antibody (NAb) titers measured by pseudovirus. **(B,C)**. Neutralizing antibody titers against ancestral (B), Delta strain (C) of live severe acute respiratory syndrome coronavirus 2 (SARS‑CoV‑2) virus. **(D)** Seral cross‐reactivity for Delta strain was expressed as fold change. The NAb concentrations were expressed as 50% inhibitory dilution (EC50) of serum. *N* = 10 per group, one spot represents one sample. Bars represent the geomean ± geometric SD, ns: *p* > 0.05, *p < 0.05, **p < 0.01, ***p < 0.001, ****p < 0.0001

To evaluate the serum‐neutralizing activity of the vaccinated mice, we measured the NAb titers against pseudovirus^31^ and live SARS‐CoV‐2 variants. As a result, 4/10 of the PS‐RBD group and 3/10 of the RNA‐RBD group could effectively neutralize the ancestral strain (the titer was > 1:4 after the first dose); while 14 days after the second dose, the GMT of NAb titers against ancestral strain increased significantly, reaching 2037 (95% CI: 1096–3802) for group 2 × PS‐RBD and 1321 (95% CI: 617–2818) for group 2 × RNA‐RBD (Figure 3B). Similar results were obtained in the pseudovirus NAb assay (Figure 3A). The GMTs of the NAb titers against the Delta strain induced by 2 × PS‐RBD (GMTs: 402, 95% CI: 269–589) decreased by 5.07‐fold compared to that against the ancestral strain, while that in the 2 × RNA‐RBD groups decreased by 2.73‐fold (Figure 3C,D).

Interestingly, though no elevation was found in binding antibody titers in heterologous regimens compared with homologous regimens, the serum NAbs level against ancestral and Delta strain in the PS‐RBD > RNA‐RBD group was higher than that in 2 × RNA‐RBD (ancestral: *p* = 0.0030; Delta: *p* = 0.1128), 2 × PS‐RBD (ancestral: *p* = 0.0088; Delta: *p* = 0.0084), and RNA‐RBD > PS‐RBD (ancestral: *p* < 0.0001; Delta: *p* = 0.0005), with GMT of 6001 (95% CI: 3548–10,233) against ancestral strain and 1306 (95% CI: 708–2455) against Delta strain. Thus, compared to the homologous regimens, the heterologous prime‐boost regimen PS‐RBD > RNA‐RBD improved the serum‐neutralizing activity to a certain extent (Figure 3B–D).

### 2.3 Antibody‐dependent cell‐mediated cytotoxicity and T‐cell immune response elicited by homologous and heterologous immunization with PS‐RBD and RNA‐RBD in mice

**FIGURE 4:**
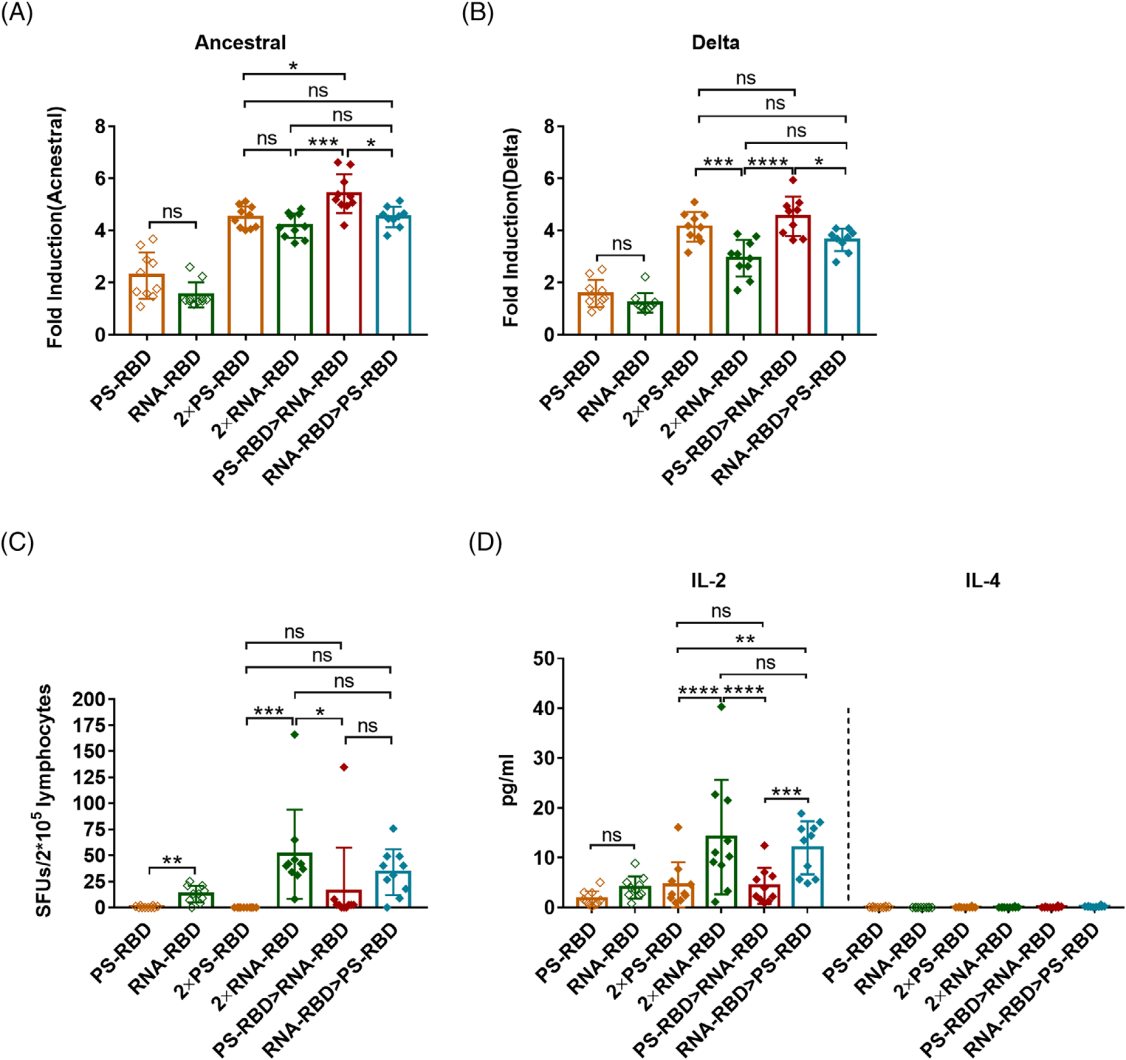
Antibody‐dependent cell‐mediated cytotoxicity (ADCC) **and severe acute respiratory syndrome coronavirus 2 (SARS‑CoV‑2) spike specific T‐cell responses for homologous and heterologous prime‐boost regimens**. **(A,B)**. ADCC effect of the immunized mouse serum against SARS‐CoV‐2 D614G strain (A) and Delta strain (B) on day 28. *N* = 10 per group, one spot represents one sample. **(C,D)**. Mice were sacrificed for the measurement of T‐cell responses. Isolated lymphocytes were stimulated by a spike peptide pool, the interferon (IFN)‐γ secreting cells were quantified by enzyme‐linked immunospot (ELISpot) assay (C), and the interleukin (IL)‐2 and IL‐4 in supernatants were quantified by meso scale discovery (MSD) (D). *N* = 10 per group, one spot represents one sample. Bars represent the mean ± SD, ns: *p* > 0.05, *p < 0.05, **p < 0.01, ***p < 0.001, ****p < 0.0001

We further analyzed the antibody‐dependent cell‐mediated cytotoxicity (ADCC) activity of the immunized mouse serum against ancestral and Delta SARS‐CoV‐2 strains. ADCC activity was detected by the luciferase reporter gene assay, and its main judgment index was the fold induction (FI) of the fluorescence. Our results showed that the ADCC activity against the ancestral strain in 2 × PS‐RBD was comparable to 2 × RNA‐RBD, with a FI (1:12 dilution) of 4.51 and 4.19, respectively. For the Delta variant, the FI in group 2 × PS‐RBD was higher than that in 2 × RNA‐RBD (*p* = 0.0180) (Figure 4A). Notably, compared with homologous regimens, the ADCC activity of PS‐RBD > RNA‐RBD against ancestral and Delta strains was higher than that of other groups (Figure 4A,B).

To study spike‐specific T‐cell responses induced by different vaccine regimens, we collected splenocytes and stimulated them with a peptide pool covering the full length of the SARS‐CoV‐2 spike, then quantified the amount of interferon (IFN)‐γ‐secreting lymphocytes by enzyme‐linked immunospot assay (ELISpot assay). Our results showed that one dose of RNA‐RBD induced higher T‐cell immune responses than PS‐RBD (*p* = 0.06). Meanwhile, all three groups containing RNA‐RBD, such as 2 × RNA‐RBD, PS‐RBD > RNA‐RBD, and RNA‐RBD > PS‐RBD, successfully induced a T cell‐mediated immune response, with 51.10, 15.58, and 33.97 spot forming units (SFUs)/2 × 10^5^ cells, respectively (Figure 4C).

To further define the Th subtype of systemic T cell immune responses induced by different vaccines, a meso scale discovery (MSD) assay was conducted. Supernatants of splenic lymphocytes stimulated by spike peptide pool were collected for interleukin (IL)‐2 and IL‐4 measurement. IL‐2 is mainly secreted by Th1 cells, while IL‐4 is secreted by Th2 cells. IL‐2 levels were highly improved in all vaccinated groups (from 1.75 to 14.13 pg/ml) when compared to IL‐4 (0.00–0.22 pg/ml). Remarkably, 2 × RNA‐RBD and RNA‐RBD > PS‐RBD induced higher levels of IL‐2 (14.13 and 12.01 pg/ml) than 2 × PS‐RBD (4.60 pg/ml, *p* < 0.005) and PS‐RBD > RNA‐RBD (4.36 pg/ml) (*p* < 0.0005) group (Figure 4D).

Therefore, the ADCC activity of PS‐RBD > RNA‐RBD was higher than that of the homologous regimens, and S‐specific T‐cell responses could be effectively induced by heterologous prime‐boost. Furthermore, the 2 × RNA‐RBD and RNA‐RBD > PS‐RBD groups induced a better Th1‐type T‐cell immune response.

### 2.4 Two vaccines mixed in different ratios produced superior immune responses to heterologous prime‐boost immunization

**FIGURE 5:**
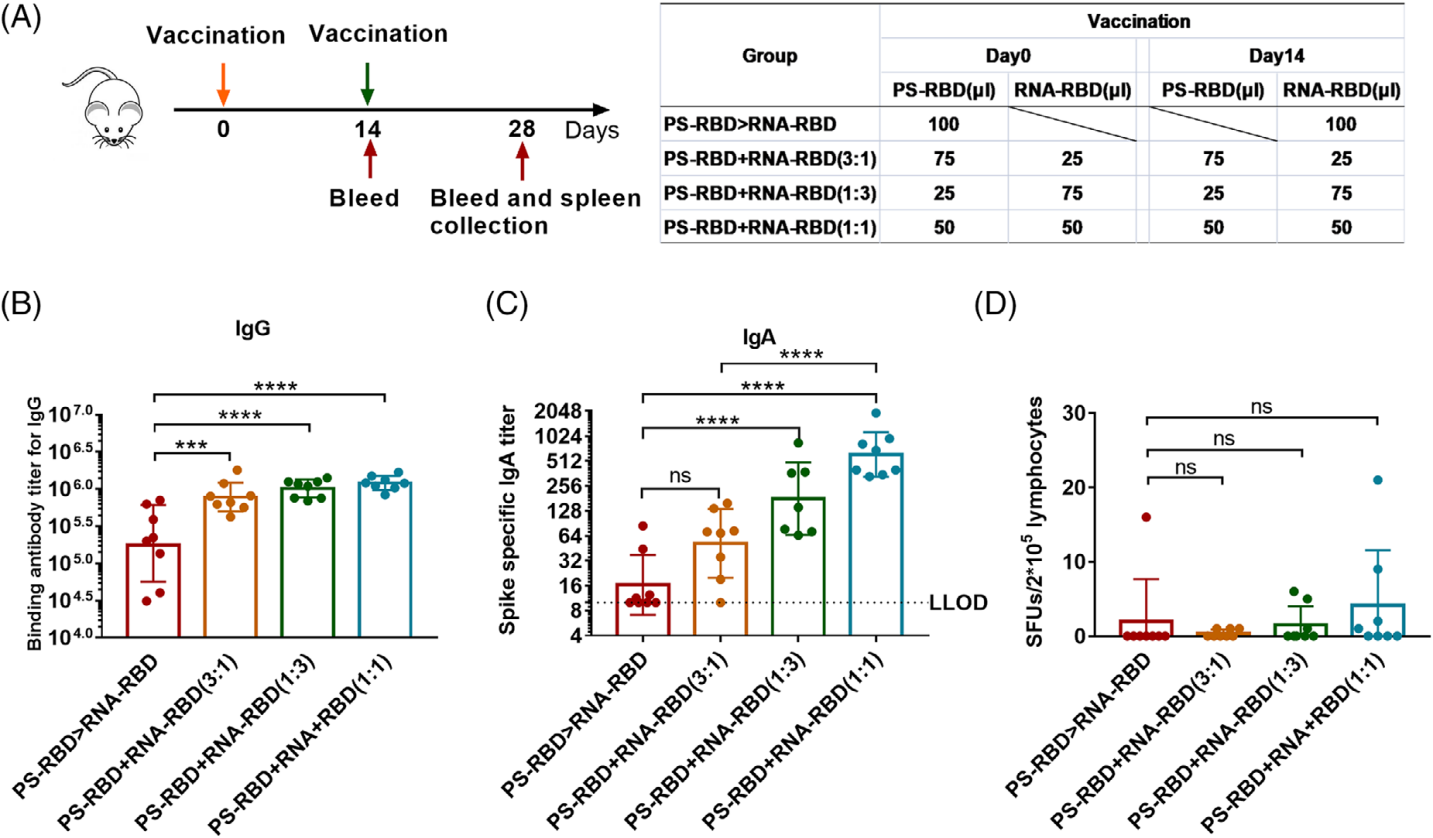
**Binding antibody and T cell responses induced by mixed formulation of PS‐RBD and RNA‐RBD in mice**. (A). Schematic representation of the experimental protocol. Mice were immunized with the mixed formulation of PS‐RBD and RNA‐RBD in different ratios: PS‐RBD+RNA‐RBD (3:1), PS‐RBD+RNA‐RBD (1:3), and PS‐RBD+RNA‐RBD (1:1) or primed with PS‐RBD and boosted with RNA‐RBD. For all mice, blood was collected on days 14 and 28. On day 28, mice were sacrificed to obtain splenocytes. (B,C). Binding antibodies response, including the Spike‐specific immunoglobulin G (IgG) (B) and IgA (C). (G). Spike‐specific T‐cell responses of splenocytes were measured by interferon (IFN)‐γ enzyme‐linked immunospot (ELISpot) assay and expressed as spot‐forming units (SFUs) per 2 × 10^5^ lymphocytes. *N* = 8 per group, one spot represents one sample. Bars represent the geomean ± geometric SD in (B,C), the mean ± SD in (D); ns: *p* > 0.05, ***p < 0.001, ****p < 0.0001

Although heterologous prime‐boost with RNA‐RBD and PS‐RBD improved immune responses but very limited, as T cells and antibodies were not boosted for the same regimen. We further mixed the two vaccines in different ratios and immunized the mice twice by 2 weeks interval, including PS‐RBD+RNA‐RBD (3:1), PS‐RBD+RNA‐RBD (1:3), and PS‐RBD+RNA‐RBD (1:1) (Figure 5A). The immune responses in mixed formulation groups were compared with that in PS‐RBD > RNA‐RBD group 2 weeks after immunization. The mixed formulation groups showed higher humoral and T‐cell immune responses. For IgG, the mixed formulation groups including PS‐RBD+RNA‐RBD (3:1) (GMTs: 78,135, 95% CI: 575,440–1,047,129), PS‐RBD+RNA‐RBD (1:3) (GMTs: 1,017,381, 95% CI: 851,138–1,230,268), PS‐RBD+RNA‐RBD (1:1) (GMTs: 1,214,185, 95% CI: 1,047,129–1,412,538) had significantly higher total IgG GMTs than PS‐RBD > RNA‐RBD (GMTs: 184,540, 95% CI: 87,096–398,107) (*p* < 0.001) (Figure 5B). A similar result was shown for IgA, the GMTs of IgA were 52 (95% CI: 28–95), 179 (95% CI: 89–355), and 611 (95% CI: 417–912) for PS‐RBD+RNA‐RBD (3:1), PS‐RBD+RNA‐RBD (1:3) and PS‐RBD+RNA‐RBD (1:1), respectively, and all mixed formulation groups were higher than PS‐RBD > RNA‐RBD (16, 95% CI: 10–28) (Figure 5C).

**FIGURE 6:**
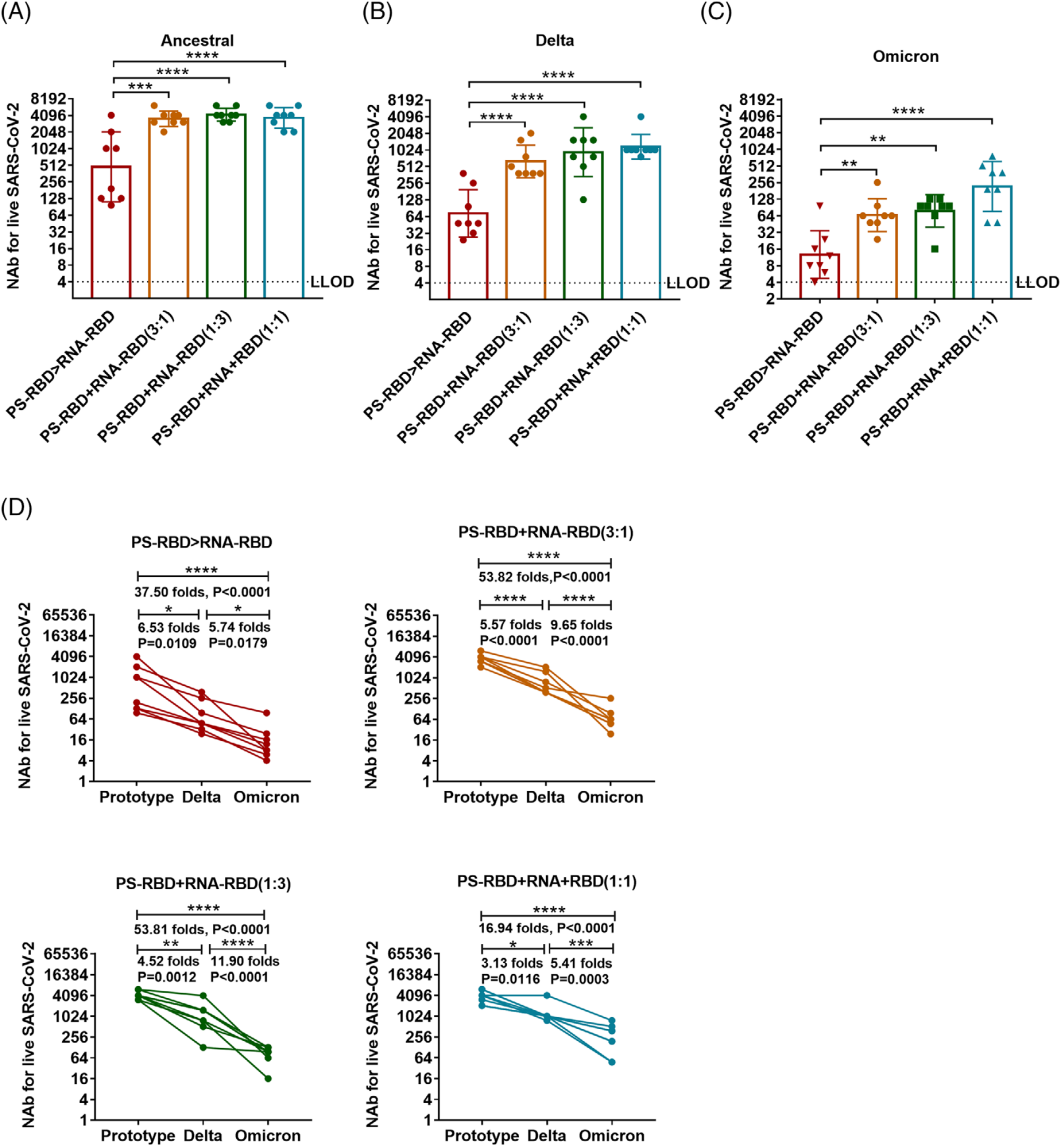
**Serum neutralizing activity induced by mixed formulation of PS‐RBD and RNA‐RBD in mice**. (A–D). The geometric mean titers (GMTs) of serum neutralizing antibodies against ancestral (A), Delta (B), and Omicron (C, D) were detected by micro cytopathogenic effect assay. Neutralizing antibody (NAb) titers were expressed as 50% effective dilution (ED50) of serum. *N* = 8 per group, one spot represents one sample. Bars represent the geomean ± geometric SD; *p < 0.05, **p < 0.01, ***p < 0.001, ****p < 0.0001

The NAb titers of the mixed immunization groups to the ancestral, Delta, and Omicron strains were significantly higher than that of the PS‐RBD > RNA‐RBD group. For the ancestral strain, the NAb GMTs induced by PS‐RBD+RNA‐RBD (3:1), PS‐RBD+RNA‐RBD (1:3), PS‐RBD+RNA‐RBD (1:1) were 3547 (95% CI: 2884–4365), 4,218 (95% CI 3548–5012), 3677 (95% CI: 2818–4898) respectively, and were significantly higher than that of PS‐RBD > RNA‐RBD (476, 95% CI: 186–1230) (*p* < 0.001) (Figure 6A). Furthermore, PS‐RBD+RNA‐RBD (1:1) showed better cross‐neutralization activity than other mixed ratios. To be specific, the NAb titers of PS‐RBD+RNA‐RBD (1:1) to Delta and Omicron decreased 3.13‐ and 5.41‐fold compared with that of PS‐RBD+RNA‐RBD (1:1) to ancestral strain. Whereas, The PS‐RBD+RNA‐RBD (3:1) decreased by 5.57‐ and 9.65‐fold, and PS‐RBD+RNA‐RBD (1:3) decreased by 4.52‐ and 11.90‐fold, respectively (Figure 6B–D).

ELISpot results showed that PS‐RBD+RNA‐RBD (3:1) induced 0.38 SFUs/2 × 10^5^ cells, PS‐RBD+RNA‐RBD (1:3) induced 1.50 SFUs/2 × 10^5^ cells, PS‐RBD+RNA‐RBD (1:1) induced 4.13 SFUs/2 × 10^5^ cells, and PS‐RBD > RNA‐RBD induced 2.00 SFUs/2 × 10^5^ cells (Figure 5D). T cell response in PS‐RBD+RNA‐RBD (1:1) was increased to some extent though without significance.

In summary, the codelivery of PS‐RBD and RNA‐RBD, especially PS‐RBD+RNA‐RBD (1:1), brought a higher level of antibody titers than PS‐RBD > RNA‐RBD and strengthened the cellular immune responses.

## 3 DISCUSSION

Different vaccine platforms induce varying immune responses. The mRNA vaccine is delivered into host cells via a lipid nanoparticle system and subsequently translated into targeting immunogens,^32^ while the protein subunit vaccine directly acts as an antigen and is ingested and processed by antigen presentation cells to induce an immune response.^33^ Thus, it's a rational attempt to combine mRNA and protein subunit vaccines for stronger and broader immune responses. In this study, an mRNA vaccine expressing RBD named RNA‐RBD and a recombinant RBD vaccine named PS‐RBD were used to optimize the vaccination strategy of mRNA and protein subunit vaccines.

PS‐RBD and RNA‐RBD induced a different modality of immune status in this study. The total IgG titer induced by one dose of PS‐RBD was 1.92 times than that induced by one dose of RNA‐RBD, but after two doses, the total binding IgG titer of the RNA‐RBD group was significantly higher than that of the PS‐RBD group. RNA‐RBD showed a stronger immune‐boosting potential in a prime‐boost vaccination procedure. In addition, heterologous immunization with PS‐RBD followed by RNA‐RBD induced a higher level of IgG response than immunization with RNA‐RBD followed by PS‐RBD (Figure 1B). Thus, PS‐RBD might be more suitable as a prime vaccine, and RNA‐RBD might be more suitable as a booster vaccine to strengthen the antibody responses. Although PS‐RBD and RNA‐RBD elicited comparable humoral responses after one or two doses of injection, RNA‐RBD induced a lower IgG1/IgG2a ratio in mice (Figure 2B). For heterologous prime‐boost regimens, the proportion of IgG2a in total IgG was improved in groups given RNA‐RBD as the prime dose, with an IgG2a ratio > 23%, whereas, IgG1 is the dominant IgG subtype in groups that were first immunized with PS‐RBD (Figure 2A). Compared with IgG1, IgG2a possesses a high affinity for the activating receptors FcγRI, FcγRIII, FcγRIV, and inhibitory receptor FcγRIIB in various myeloid cells and performs more abundant immune functions in defending against virus invasion.^34^ Therefore, immunizing with mRNA vaccine followed by protein subunit vaccine might broaden the antibody‐mediated immune response and this might subsequently assist in virus elimination in vivo. In addition, IgG2a/IgG1 might reflect the type of T cell response to some extent.^35^ In this study, Th1‐type responses indicated by IFN‐γ and IL‐2 were relatively higher in groups that received RNA‐RBD as the prime dose (RNA‐RBD, 2 × RNA‐RBD, and RNA‐RBD > PS‐RBD) (Figure 4C), which is consistent with the relatively higher level of IgG2a/IgG1 in these groups.

Studies have confirmed the positive correlation between binding antibodies and NAbs,^36^ whereas, our data in mouse models show that the correlation is not strictly proportional.^37^ In this study, although heterologous prime‐boost immunization with RNA‐RBD and PS‐RBD didn't increase the level of total IgG nor the individual IgG1, IgG2a, IgG2b, and IgG3 subtypes, it improved serum neutralizing activity against Delta variant (Figure 3B–D). Our previous results on heterologous prime‐boost immunization of various COVID‐19 vaccines showed that administration of the adenovirus vectored vaccine followed by recombinant protein vaccine or inactivated viral vaccine effectively upregulated the proportion of NAb in total binding antibody.^22^, ^37^, ^38^ In addition, Shixia Wang et al. reported that the “DNA vaccine prime, protein vaccine boost” strategy induced stronger HIV gp120‐specific antibody and B cell responses than homologous vaccination,^14^ demonstrating that the DNA vaccine is a promising vaccine platform for primary immunization. Upon, heterologous boosting with mRNA vaccines after recombinant protein vaccine priming showed higher NAb level than the “mRNA vaccine prime and protein vaccine boost” regimen. It seemed very different from the phenomenon found in other nucleic acid vaccines, like adenovirus and DNA‐vectored vaccines, which showed higher NAb titer in adenovirus or DNA‐vectored vaccine primed regimens than protein vaccine primed regimens. This might be due to the underlying differences between mRNA and DNA vaccines in antigen expression, presentation, and immune activation in vivo and more experiments need to be done to clarify it.^32^, ^39^

Due to the rapid mutation of the spike protein during the transmission of the SARS‐CoV‐2, the neutralizing activity against the prevalent virus strains induced by existing COVID‐19 vaccines of the first generation declined to varying extents.^40^, ^41^ The Omicron variant, which appeared at the end of 2021 with 38 mutations in the spike protein, has rapidly replaced other VOCs and become the dominant variant worldwide. As presented in Figure 2, heterologous prime‐boost regimen PS‐RBD > RNA‐RBD improved the vaccine‐induced NAb response against the ancestral and Delta strain, but at a modest level. The self‐adjuvant effect of the mRNA vaccine is considered a double sword. Proper self‐adjuvant is beneficial to immunogenicity, but an excessive self‐adjuvant effect will influence the immunogenicity of the mRNA vaccine by affecting the immunogen translation and expression.^39^, ^42^, ^43^ In our study, co‐delivery of mRNA and protein vaccines could obtain the self‐adjuvant effect of mRNA vaccine, while also ensuring the absolute amount of immunogen processed by dendritic cells, and showed to dramatically elevate the level of NAb against ancestral, Delta, and Omicron (Figure 6). A similar phenomenon was also observed by Li et al. using a co‐delivery strategy of DNA and protein.^23^ Besides the integrated advantage mentioned above, both mRNA and DNA vaccines express the targeting antigen in vivo. Thus, they could imitate the process of natural SARS‐CoV‐2 virus invasion and display more conformational epitopes than recombinant proteins. Co‐delivery with recombinant proteins might induce antibodies against more broadly neutralizing epitopes. In addition, endogenous vaccines could promote both major histocompatibility complex I (MHC‐I) and MHC‐II antigen presentation and mediate a better T cell immune response, which makes up for the deficiency of recombinant protein vaccine in T cell immunity.

Taken together, we investigated the proper combined immunization strategy for the novel mRNA and recombinant protein subunit COVID‐19 vaccine candidates in a mouse model. We described that the heterologous prime‐boost regimen “PS‐RBD prime and RNA‐RBD boost” could enhance the neutralizing and ADCC activity induced by PS‐RBD and RNA‐RBD to a certain amount but with a limited Th1 cellular response. Interestingly, the mixed formulation of PS‐RBD and RNA‐RBD achieved a stronger and broader immune response by integrating the advantages of the two divergent forms of the vaccine. To be specific, the mixed formulation markedly improved the cross‐NAb responses against ancestral, Delta, and Omicron strains and elicited a more robust Th1‐type response than heterologous priming with PS‐RBD and boosting with RNA‐RBD. This study pointed out the importance of the vaccination order and formulation of mRNA and protein vaccines on immune response in a mouse model, but it should be noted that murine data do not always translate to humans, further investigations in non‐human primates and human beings are required. Nevertheless, the stability of the mixed formulation vaccine and the duration of the vaccine‐induced immune responses need to be further investigated.

## 4 MATERIALS AND METHODS

### 4.1 Animals and vaccines

Specific pathogen‐free (SPF) grade BALB/c female mice (6–8 weeks old, 18–22 g) were provided by the National Institute for Food and Drug Control. RNA‐RBD used in this study was an mRNA vaccine expressing the receptor binding domain of ancestral SARS‐CoV‐2. PS‐RBD was a protein subunit vaccine with a dimeric RBD as the immunogen and Alum as the adjuvant. In Figures 1, 2, 3, 4, mice were immunized with 100 μl PS‐RBD (10 μg, 1/5 of high dose for humans) or 100 μl RNA‐RBD (5 μg, 1/5 of high dose for humans) on day 0 and day 14. For groups shown in Figure 5A, mice were immunized with 100 μl (5 μg, 1/5 of low dose for humans) PS‐RBD on day 0 followed by 100μlRNA‐RBD (3 μg, 1/5 of low dose for humans) on day 14 for PS‐RBD > RNA‐RBD; for the mixed formulation groups, PS‐RBD and RNA‐RBD were mixed by different ratios and then administered in mice at once. To be specific, mice were immunized with 3.75 μg (75 μl) PS‐RBD mixed with 0.75 μg (25 μl) RNA‐RBD for PS‐RBD+RNA‐RBD (3:1), 1.25 μg PS‐RBD (25 μl) mixed with 2.25 μg (75 μl) RNA‐RBD for PS‐RBD+RNA‐RBD (1:3) and 2.50 μg (50 μl) PS‐RBD mixed with 1.50 μg (50 μl) RNA‐RBD for PS‐RBD+RNA‐RBD (1:1) on day 0 and day 14, respectively. All mice were randomly grouped before vaccination. All the vaccines were injected intramuscularly into the hind legs.

### 4.2 Enzyme‐linked immunosorbent assay for estimating spike‐specific IgG and IgA titers

Spike‐specific binding antibodies were assayed by enzyme‐linked immunosorbent assay: 96‐well EIA/RIA plates were coated with 1 μg of spike protein at 4°C overnight. The plates were washed three times with PBST (phosphate‐buffered saline [PBS] containing 0.05% Tween‐20) and then blocked using PBS with 10% fetal bovine serum and 5% sucrose for 2 h at 37°C. After incubation, plates were washed three times again with PBST and added to 10‐fold serially‐diluted test samples, and incubated for 37°C at 1 h. After incubation, plates were washed five times with PBST and added to a 1:10000 dilution of HRP‐labeled goat anti‐mouse IgG, IgG1, IgG2a, IgG2b, IgG3 or IgA secondary antibody (ZSGB‐BIO, cat#ZB2305; Abcam, Cambridge, UK: ab97235, ab97240, ab97245, ab97246, and ab97260). After 1 h of incubation at 37°C, the absorbance of the plates was detected with the substrate (WantaiBioPharm, cat#N20200722) at wavelengths of 450 and 630 nm. The endpoint of serum antibody titers was determined as the reciprocal of the highest dilution that was 2.1‐fold higher than the optical absorbance value of the negative control.

### 4.3 Serum NAb detection using pseudovirus

The neutralization capacity of the pseudo‐virus (Wuhan‐hu‐1, GenBank: MN908947, optimized for human cell expression) was examined following a previous protocol.^31^

Pseudovirus (10^4^ 50% tissue culture infective dose, 50 μl) were co‐cultured with an equal volume of 3‐fold gradient diluted serum for 1 h at 37°C, and then 2 × 10^4^/well Huh‐7 cells were added in 96‐well plates at 100 μl/well and incubated at 37°C with 5% CO2 for 20–28 h. After incubation, 100 μl of chemiluminescence detection reagent (PerkinElmer cat#6‐66769) was added to each well, and the substrate was mixed and transferred to the luminescence plate (XIAMEN YJM LABWARE CO. LTD. Cat#KH 25). The Reed‐Muench method was used to calculate the virus neutralization titer.

### 4.4 Serum NAb detection using live‐virus

The micro cytopathogenic effect assay was used for the measurement of serum NAbs against live SARS‐CoV‐2.^44^ The ancestral strain SARS‐CoV‐2/human/CHN/CN1/2020 (GenBank: MT407649.1),^45^ Delta (B.1.617.2) strain hCoV‐19/China/JS07/2021 (GISAID EPI_ISL_4515846)^46^ and Omicron BA.1 (B.1.1.529.1) strain isolated in China were used for NAb detection. Serum samples were inactivated at 56°C for 30 min and then serially diluted with cell culture medium in a 2‐fold dilution gradient. The diluted serum samples were incubated with equal volumes of live SARS‐CoV‐2 suspension (100 50% cell culture infective dose, 50 μl) for 2 h at 37·0°C. Vero E6 cells (1.0 × 10⁵ to 2.0 × 10⁵ cells per ml) were then added to the serum–virus suspensions in microplates and incubated at 36.5°C for 5 days. Cytopathic effects were recorded under microscopes, and NAb titers were calculated by the number of dilutions under 50% protective conditions. The results were expressed as GMT with the lowest dilution gradient of 1:4.

### 4.5 IFN‐γ ELISpot assay

Mouse IFN‐γ ELISpot kit (BD, cat#551083) was used for IFN‐γ enzyme‐linked immunosorbent spot (ELISpot) assay. Mouse splenocytes (2 × 10^5^ cells/well) were collected and co‐cultured with a peptide pool spanning the SARS‐CoV‐2 spike protein at 37°C in 5% CO_2_ for 20 h. A peptide pool was generated as follows: a panel of consecutive 15‐mer peptides overlapped with 9 amino acids were synthesized spanning the whole spike protein and pooled by the concentration of 5 μg/ml for each peptide. The supernatant was removed and IFN‐γ positive cells were detected using the antibody from the kit. Counts were performed with an ELISpot reader (ChampSpot III ELISpot Reader; Saizhi, Beijing, China) and the final result was obtained after subtracting the background value from the raw value.

### 4.6 Antibody‐dependent cell‐mediated cytotoxicity

Jurkat cells stably expressing murine FcRγIII and fluorophore reporter genes were used as effector cells (Promega, CS1779B06), and 293T cells transfected with pcDNA3.1‐spike and transiently expressing SARS‐CoV‐2 spike were prepared as target cells before ADCC measurement. For experiments, serum samples were diluted with a 2.5‐fold gradient and incubated with 4.25 × 10^3^ Jurkat‐mFcRγIII cells and 7.5 × 10^4^ target cells together for 16 h at 37°C. After incubation, PerkinElmer (Waltham, MA) was added for color development, and relative light units (RLUs) were detected using a Micro2000. The FI was calculated by FI = RLU (induction − background)/RLU (negative serum control − background).

### 4.7 Multiple cytokine assays

Mouse splenocytes (1 × 10^6^ per well) were stimulated by the peptide pools spanning the SARS‐CoV‐2 spike protein at a concentration of 5 μg/ml for each peptide. Supernatants were collected after 20 h of culturing. Cytokines secreted by splenocytes were detected by a V‐PLEX Proinflammatory Panel 1 (mouse) Kit (MSD, cat# K15048D‐1), and the final result was calculated by the experimental group minus background.

### 4.8 Statistical analysis

After antibody titers were log‐transformed, comparisons between two groups of data were analyzed by student *t*‐test. The one‐way ANOVA test was used for comparisons of more than two groups. *p*‐Values < 0.05 were considered significant. All statistical analyses were conducted using GraphPad Prism 7.0 (GraphPad Software, Inc).

## AUTHOR CONTRIBUTIONS

ZL, JW, MX and QH conceived this study. QH designed the study. QH, JZ and XY performed the majority of the experiments and analyzed the data. JZ wrote the original draft. QH reviewed and revised the original draft. JL, YB, CA, BC, QM and FG helped analyze the data and conduct some experiments. All authors reviewed and approved the manuscript.

## CONFLICT OF INTEREST

The authors declare that they have no conflict of interest.

5

## ETHICS STATEMENT

All animal research protocols were approved by the Institutional Animal Care and Use Committee at National Institutes for Food and Drug Control, China (No. 2022‐B016), and these protocols were conducted in accordance with regulations regarding the management of laboratory animals (National Science and Technology Commission no. 2 of Oct. 31, 1988) and “guidance notes on the treatment of experimental animals” (Chinese version (2006) no. 398). All institutional guidelines for animal care and use were strictly followed throughout the study.

## Data Availability

All data in this study are available from the corresponding author on reasonable request.

## References

[1] Su S , Du L , Jiang S . Learning from the past: development of safe and effective COVID‐19 vaccines. Nat Rev Microbiol. 2021;19(3):211‐219. 33067570 10.1038/s41579-020-00462-y PMC7566580

[2] Team VGCVT . COVID19 vaccine tracker. 2022 Accessed October 28, 2022. https://covid19.trackvaccines.org/

[3] Tanriover MD , Doğanay HL , Akova M , et al. Efficacy and safety of an inactivated whole‐virion SARS‐CoV‐2 vaccine (CoronaVac): interim results of a double‐blind, randomized, placebo‐controlled, phase 3 trial in Turkey. The Lancet. 2021;398(10296):213‐222. 10.1016/S0140-6736(21)01429-X PMC8266301 34246358

[4] Sadoff J , Gray G , Vandebosch A , et al. Safety and efficacy of single‐dose Ad26.COV2.S vaccine against COVID‐19. N Engl J Med. 2021;384(23):2187‐2201. 33882225 10.1056/NEJMoa2101544 PMC8220996

[5] Kennedy RB . Efficacy of an adenovirus type 5 vectored SARS‐CoV‐2 vaccine. The Lancet. 2022;399(10321):212‐213. 10.1016/S0140-6736(21)02834-8 PMC8700275 34953524

[6] Ella R , Reddy S , Blackwelder W , et al. Efficacy, safety, and lot‐to‐lot immunogenicity of an inactivated SARS‐CoV‐2 vaccine (BBV152): interim results of a randomised, double‐blind, controlled, phase 3 trial. The Lancet. 2021;398(10317):2173‐2184. 10.1016/S0140-6736(21)02000-6 PMC8584828 34774196

[7] Dai L , Gao L , Tao L , et al. Efficacy and safety of the rbd‐dimer‐based COVID‐19 vaccine ZF2001 in adults. N Engl J Med. 2022;386(22):2097‐2111. 35507481 10.1056/NEJMoa2202261 PMC9127771

[8] Polack FP , Thomas SJ , Kitchin N , et al. COVID‐19 vaccine breakthrough infections reported to CDC — United States, January 1–April 30, 2021. N Engl J Med. 2020;383(27):2603‐2615.33301246

[9] Bergwerk M , Gonen T , Lustig Y , et al. COVID‐19 breakthrough infections in vaccinated health care workers. N Engl J Med. 2021;385(16):1474‐1484. 34320281 10.1056/NEJMoa2109072 PMC8362591

[10] Lu S . Heterologous prime‐boost vaccination. Curr Opin Immunol. 2009;21(3):346‐351. 19500964 10.1016/j.coi.2009.05.016 PMC3743086

[11] Wang S , Parker C , Taaffe J , Solórzano A , García‐Sastre A , Lu S . Heterologous HA DNA vaccine prime–inactivated influenza vaccine boost is more effective than using DNA or inactivated vaccine alone in eliciting antibody responses against H1 or H3 serotype influenza viruses. Vaccine. 2008;26(29‐30):3626‐3633. 18538900 10.1016/j.vaccine.2008.04.073 PMC2802517

[12] Excler JL , Kim JH . Novel prime‐boost vaccine strategies against HIV‐1. Expert Rev Vaccin. 2019;18(8):765‐779. 10.1080/14760584.2019.1640117 31271322

[13] Aleshin SE , Timofeev AV , Khoretonenko MV , et al. Combined prime‐boost vaccination against tick‐borne encephalitis (TBE) using a recombinant vaccinia virus and a bacterial plasmid both expressing TBE virus non‐structural NS1 protein. BMC Microbiol. 2005;5:45. 16076390 10.1186/1471-2180-5-45 PMC1187892

[14] Wang S , Arthos J , Lawrence JM , et al. Enhanced immunogenicity of gp120 protein when combined with recombinant DNA priming to generate antibodies that neutralize the JR‐FL primary isolate of human immunodeficiency virus type 1. J Virol. 2005;79(12):7933‐7937. 15919951 10.1128/JVI.79.12.7933-7937.2005 PMC1143658

[15] Burm R , Maravelia P , Ahlen G , et al. Novel prime‐boost immune‐based therapy inhibiting both hepatitis B and D virus infections. Published online: August 17, 2022. 10.1136/gutjnl-2022-327216 PMC10176361 35977815

[16] Liu X , Shaw RH , Stuart ASV , et al. Safety and immunogenicity of heterologous versus homologous prime‐boost schedules with an adenoviral vectored and mRNA COVID‐19 vaccine (Com‐COV): a single‐blind, randomised, non‐inferiority trial. The Lancet. 2021;398(10303):856‐869. 10.1016/S0140-6736(21)01694-9 PMC8346248 34370971

[17] Kaku CI , Champney ER , Normark J , et al. Broad anti–SARS‐CoV‐2 antibody immunity induced by heterologous ChAdOx1/mRNA‐1273 vaccination. Science. 2022;375:1041‐1047. 35143256 10.1126/science.abn2688 PMC8939765

[18] Barros‐Martins J , Hammerschmidt SI , Cossmann A , et al. Immune responses against SARS‐CoV‐2 variants after heterologous and homologous ChAdOx1 nCoV‐19/BNT162b2 vaccination. Nature medicine. 2021;27(9):1525‐1529. 10.1038/s41591-021-01449-9 PMC8440184 34262158

[19] Xu X , Liao Y , Jiang G , et al. Immunological evaluation of an mRNA vaccine booster in individuals fully immunized with an inactivated SARS‐CoV‐2 vaccine. Clin Transl Med. 2022;12(6):e875. 35678117 10.1002/ctm2.875 PMC9178391

[20] Campos GRF , Almeida NBF , Filgueiras PS , et al. Booster dose of BNT162b2 after two doses of CoronaVac improves neutralization of SARS‐CoV‐2 Omicron variant. Commun Med. 2022;2:76. 35784447 10.1038/s43856-022-00141-4 PMC9242982

[21] Cohen G , Jungsomsri P , Sangwongwanich J , et al. Immunogenicity and reactogenicity after heterologous prime‐boost vaccination with CoronaVac and ChAdox1 nCov‐19 (AZD1222) vaccines. Hum Vaccin Immunother. 2022;18(5):2052525. 35323079 10.1080/21645515.2022.2052525 PMC9115782

[22] He Q , Mao Q , An C , et al. Heterologous prime‐boost: breaking the protective immune response bottleneck of COVID‐19 vaccine candidates. Emerg Microbes Infect. 2021;10(1):629‐637. 33691606 10.1080/22221751.2021.1902245 PMC8009122

[23] Li Y , Bi Y , Xiao H , et al. A novel DNA and protein combination COVID‐19 vaccine formulation provides full protection against SARS‐CoV‐2 in rhesus macaques. Emerg Microbes Infect. 2021;10(1):342‐355. 33555988 10.1080/22221751.2021.1887767 PMC7928010

[24] WHO . COVID‐19 ‐ Landscape of novel coronavirus candidate vaccine development worldwide. 2022 Accessed October 28, 2022. https://www.who.int/publications/m/item/draft‐landscape‐of‐covid‐19‐candidate‐vaccines

[25] Fang E , Liu X , Li M , et al. Advances in COVID‐19 mRNA vaccine development. Signal Transduct Target Ther. 2022;7(1):94. 35322018 10.1038/s41392-022-00950-y PMC8940982

[26] Tartof SY , Slezak JM , Puzniak L , et al. Durability of BNT162b2 vaccine against hospital and emergency department admissions due to the omicron and delta variants in a large health system in the USA: a test‐negative case–control study. Lancet Respir Med. 2022;10(7):689‐699. 35468336 10.1016/S2213-2600(22)00101-1 PMC9033225

[27] Kaplonek P , Fischinger S , Cizmeci D , et al. mRNA‐1273 vaccine‐induced antibodies maintain Fc effector functions across SARS‐CoV‐2 variants of concern. Immunity. 2022;55(2):355‐365.e354. 35090580 10.1016/j.immuni.2022.01.001 PMC8733218

[28] Zhao X , Zhang R , Qiao S , et al. Omicron SARS‐CoV‐2 neutralization from inactivated and ZF2001 vaccines. New Engl J Med. 2022;387(3):277‐280. 35793198 10.1056/NEJMc2206900 PMC9342420

[29] Shinde V , Bhikha S , Hoosain Z , et al. Efficacy of NVX‐CoV2373 COVID‐19 vaccine against the B.1.351 variant. N Engl J Med. 2021;384(20):1899‐1909. 33951374 10.1056/NEJMoa2103055 PMC8091623

[30] Ramasamy M , Heath P , Green C , et al. A single‐blind, randomised, phase II UK multi‐centre study to determine reactogenicity and immunogenicity of heterologous prime/boost COVID‐19 vaccine schedules – Stage 2. 2021.

[31] Li Q , Wu J , Nie J , et al. The impact of mutations in SARS‐CoV‐2 spike on viral infectivity and antigenicity. Cell. 2020;182(5):1284‐1294.e1289. 32730807 10.1016/j.cell.2020.07.012 PMC7366990

[32] Pardi N , Hogan MJ , Porter FW , Weissman D . mRNA vaccines ‐ a new era in vaccinology. Nat Rev Drug Discov. 2018;17(4):261‐279. 29326426 10.1038/nrd.2017.243 PMC5906799

[33] Pollet J , Chen WH , Strych U . Recombinant protein vaccines, a proven approach against coronavirus pandemics. Adv Drug Deliv Rev. 2021;170:71‐82. 33421475 10.1016/j.addr.2021.01.001 PMC7788321

[34] Bruhns P . Properties of mouse and human IgG receptors and their contribution to disease models. Blood. 2012;119(24):5640‐5649. 22535666 10.1182/blood-2012-01-380121

[35] Kanai N , Min WP , Ichim TE , Wang H , Zhong R . Th1/Th2 xenogenic antibody responses are associated with recipient dendritic cells. Microsurgery. 2007;27(4):234‐239. 17477419 10.1002/micr.20342

[36] Manenti A , Gianchecchi E , Dapporto F , et al. Evaluation and correlation between SARS‐CoV‐2 neutralizing and binding antibodies in convalescent and vaccinated subjects. J Immunol Methods. 2022;500:113197. 34843712 10.1016/j.jim.2021.113197 PMC8619878

[37] He Q , Mao Q , Zhang J , et al. Heterologous immunization with adenovirus vectored and inactivated vaccines effectively protects against SARS‐CoV‐2 variants in mice and macaques. Front Immunol. 2022;13:949248. 10.3389/fimmu.2022.949248 PMC9428284 36059554

[38] He Q , Mao Q , Peng X , et al. Immunogenicity and protective efficacy of a recombinant protein subunit vaccine and an inactivated vaccine against SARS‐CoV‐2 variants in non‐human primates. Signal Transduct Target Ther. 2022;7(1):69. 35241645 10.1038/s41392-022-00926-y PMC8892123

[39] Wang Q , Song Z , Yang J , et al. Transcriptomic analysis of the innate immune signatures of a SARS‐CoV‐2 protein subunit vaccine ZF2001 and an mRNA vaccine RRV. Emerg Microbes Infect. 2022;11(1):1145‐1153. 35343384 10.1080/22221751.2022.2059404 PMC9037177

[40] Shen X . Boosting immunity to Omicron. Nat Med. 2022;28(3):445‐446. 35210597 10.1038/s41591-022-01727-0

[41] Bian L , Liu J , Gao F , et al. Research progress on vaccine efficacy against SARS‐CoV‐2 variants of concern. Hum Vaccin Immunother. 2022;18(5):2057161. 35438600 10.1080/21645515.2022.2057161 PMC9115786

[42] Linares‐Fernández S , Lacroix C , Exposito JY , Verrier B . Tailoring mRNA vaccine to balance innate/adaptive immune response. Trends in molecular medicine. 2020;26(3):311‐323. 31699497 10.1016/j.molmed.2019.10.002

[43] Kowalczyk A , Doener F , Zanzinger K , et al. Self‐adjuvanted mRNA vaccines induce local innate immune responses that lead to a potent and boostable adaptive immunity. Vaccine. 2016;34(33):3882‐3893. 27269061 10.1016/j.vaccine.2016.05.046

[44] Wu Z , Hu Y , Xu M , et al. Safety, tolerability, and immunogenicity of an inactivated SARS‐CoV‐2 vaccine (CoronaVac) in healthy adults aged 60 years and older: a randomised, double‐blind, placebo‐controlled, phase 1/2 clinical trial. Lancet Infect Dis. 2021;21(6):803‐812. 33548194 10.1016/S1473-3099(20)30987-7 PMC7906628

[45] Zhang Y , Zeng G , Pan H , et al. Safety, tolerability, and immunogenicity of an inactivated SARS‐CoV‐2 vaccine in healthy adults aged 18–59 years: a randomised, double‐blind, placebo‐controlled, phase 1/2 clinical trial. Lancet Infect Dis. 2021;21(2):181‐192. 33217362 10.1016/S1473-3099(20)30843-4 PMC7832443

[46] Li J , Hou L , Guo X , et al. Heterologous AD5‐nCOV plus CoronaVac versus homologous CoronaVac vaccination: a randomized phase 4 trial. Nat Med. 2022;28(2):401‐409. 35087233 10.1038/s41591-021-01677-z PMC8863573

